# Assessment of intratumor immune-microenvironment in colorectal cancers with extranodal extension of nodal metastases

**DOI:** 10.1186/s12935-018-0634-8

**Published:** 2018-09-06

**Authors:** Matteo Fassan, Luca Vianello, Diana Sacchi, Giuseppe N. Fanelli, Giada Munari, Marco Scarpa, Rocco Cappellesso, Fotios Loupakis, Cristiano Lanza, Roberta Salmaso, Claudia Mescoli, Nicola Valeri, Marco Agostini, Edoardo D’Angelo, Sara Lonardi, Salvatore Pucciarelli, Nicola Veronese, Claudio Luchini, Massimo Rugge

**Affiliations:** 1 Surgical Pathology & Cytopathology Unit, Department of Medicine (DIMED), University of Padua, via Gabelli 61, 35121 Padua, Italy; 2 Department of Surgical Oncology and Gastroenterology (DiSCOG), University of Padua, Padua, PD Italy; 3 Unit of Oncology 1, Department of Clinical and Experimental Oncology, Istituto Oncologico Veneto, IOV-IRCCS, Padua, PD Italy; 4 Division of Molecular Pathology, The Institute of Cancer Research, Sutton, London, UK; 5 Department of Medicine, The Royal Marsden NHS Trust, Sutton, London, UK; 6 Nanoinspired Biomedicine Laboratory, Institute of Pediatric Research, Fondazione Città della Speranza, Padua, PD Italy; 7 Department of Nanomedicine, The Methodist Hospital Research Institute, Houston, TX USA; 8 National Research Council, Neuroscience Institute, Aging Branch, Padua, PD Italy; 9 Department of Diagnostics and Public Health, Section of Pathology, University and Hospital Trust of Verona, Verona, VR Italy; 10 Veneto Cancer Registry, Padua, PD Italy; 11 National Institute of Gastroenterology-Research Hospital, IRCCS “S. de Bellis”, 70013 Castellana Grotte, BA Italy

**Keywords:** Extranodal extension, Metastasis, Colorectal cancer, Biomarkers

## Abstract

**Background:**

No data is available on the molecular background of the extra-nodal extension (ENE) of lymph node metastasis (LN) in colorectal cancer (CRC).

**Methods:**

A series of 22 ENE-positive CRCs was considered and three samples per case were selected (the primary CRC, an ENE-negative and an ENE-positive metastatic LN). Samples (n = 66) were analysed by immunohistochemistry for PD-L1, CD4, CD8, CD68 and CD80. Fifteen out of twenty-two cases were further profiled through a hotspot multigene mutational custom panel, including 164 hotspot regions of *AKT1*, *APC*, *BRAF*, *CTNNB1*, *KIT*, *KRAS*, *NRAS*, *PDGFRA*, *PIK3CA*, *PTEN* and *TP53* genes.

**Results:**

A significantly higher percentage of CD4-, CD8- and CD68-positive cells was observed at the invasive front of both CRCs and in ENE in contrast with what observed at the core of both CRCs and their matched nodal metastases. ENE was also characterized by a significantly higher number of CD80-positive cells. No significant difference was observed in PD-L1 distribution among the different specimens. Fourteen out of 15 CRCs (93%) showed at least a driver mutation. The most frequently mutated gene was *TP53* (n = 8 tumors), followed by *APC* (n = 6), *BRAF* (n = 4), *KRAS*, *NRAS* and *PIK3CA* (n = 2). In 11 out of 15 CRCs (73%) the mutational profiling of the primary tumor was consistent with what obtained from the two matched LNs.

**Conclusions:**

A heterogeneous intratumor immune-microenvironment has been observed in ENE-positive CRCs, which are characterized by an increased leukocytic infiltration at the ENE invasive front.

## Background

Colorectal cancer (CRC) is the third more frequent cancer and the fourth cause of cancer death all over the world [1].

CRC survival is mainly dependent upon the tumor-node-metastasis (TNM) stage of disease at diagnosis [1, 2]. However, not all the patients, who have the same stage, show comparable prognoses. Therefore, further research is needed to identify novel prognostic factors for the stratification of risk of recurrence in patients classified with the same stage of disease.

Recently, the extra-nodal extension (ENE) of nodal metastasis has emerged as a promising and independent prognostic factor in several epithelial malignancies [3–14].

From the pathological point of view, ENE is different from free tumor deposits in the fibroadipose tissue, even if both conditions present similar phenotypic features. In contrast to what is observed in ENE, tumor deposits do not present residual nodal tissue and are considered as a separate entity in the tumor-node-metastasis (TMN) staging system, having a specific category into the N group (i.e. N1c) [15]. On the other hand, ENE, which is defined by an invasion of cancer cells/glands from an intra-nodal metastasis through the lymph node capsule, is still not considered in the TNM system as a prognostic parameter in nodal-metastatic CRC patients.

In a recent meta-analysis from our group, specifically designed on the prognostic role of ENE in CRC [16], we demonstrated the importance of such parameter in influencing the survival of CRC patients. Notably, 611 patients out of the analysed nodal-positive 1336 (45.7%), showed an ENE-positive status, Indicating that ENE is a frequent condition among CRC metastatic tumors.

The invading potential of tumor cells through the LN capsule may reflect the aggressiveness acquired from a clone deriving from the primary tumor or occurring within the metastatic nodes [17]. Therefore, several authors have proposed the detection and quantification of ENE in the surgical specimen as a novel diagnostic tool for a correct assessment of tumor staging and for a personalization of adjuvant therapy [16]. However, no information is available on the molecular mechanisms underpinning the ENE phenotype. Thus, the main aim of this work was the investigation of the heterogeneous intratumor molecular landscape and immune-microenvironment in a series of ENE-positive CRCs.

## Materials and methods

### Patients and samples

A series of consecutive 65 TNM stages III (*n *= 45) and IV (*n *= 20) formalin-fixed paraffin embedded (FFPE) primary CRC and their matched LNs were retrieved from the archives of the Surgical Pathology Unit of the Padua University (samples collected in year 2016). The study was approved by the local Ethic Committee (n. 0032705/16).

Patients who had received neoadjuvant chemo-radiotherapy, patients under the age of 20, and cases with a number of histologically examined LNs lower than 12 were excluded from the selection.

Two gastrointestinal pathologists, who were unaware of any clinical information, re-evaluated all the original haematoxylin and eosin stained slides according to the 2010 World Health Organization classification [18]. The presence of ENE was defined as an extension of tumor cells through the nodal capsule into the perinodal adipose tissue [5] and it was assessed as present or absent. Tumor cell nodules in perivisceral adipose tissue, which are not connected with the primary tumor and are not surrounded by lymphoid tissue, were excluded due to a consideration of them as tumor deposits.

Only cases with adequate material for the subsequent molecular characterization were selected. In each case three different matched samples were obtained: (i) the primary CRC; (ii) a metastatic LN with no extranodal extension of the tumor (i.e. ENE-negative LNs were entirely examined by serial sections to exclude the present of extra-nodal involvement); (iii) an ENE positive metastatic LN.

A total of 26 cases resulted ENE positive, but adequate material was available only for 22 (M/F = 15/7; age 70.2 ± 13.7, range 40–89; grading G2 = 4, G3 = 18; staging IIIb = 2, IIIc = 14, IV = 6; all Caucasian; DNA mismatch repair machinery deficient tumors [MMRd] = 4, all with defects in MLH1/PMS2 expression). Thus, a total of 66 samples (i.e. three per case) were selected and further analysed.

### Immunohistochemistry

Immunohistochemical stains for PD-L1 (clone 22C3; Dako, Glostrup, Denmark), CD4 (Dako), CD8 (Dako), CD68 (Dako), CD80 (Novus Biologicals, Littleton, CO), MLH1 (ES05; Dako), MSH2 (clone FE11; Dako), MSH6 (clone EP49; Dako), and PMS2 (clone EP51; Dako) were automatically performed in 3–4 μm sections using the Bond Polymer Refine Detection kit (Leica Biosystems, Newcastle upon Tyne, UK) in the BOND-MAX system (Leica Biosystems). Appropriate positive and negative controls were run concurrently.

**Fig. 1:**
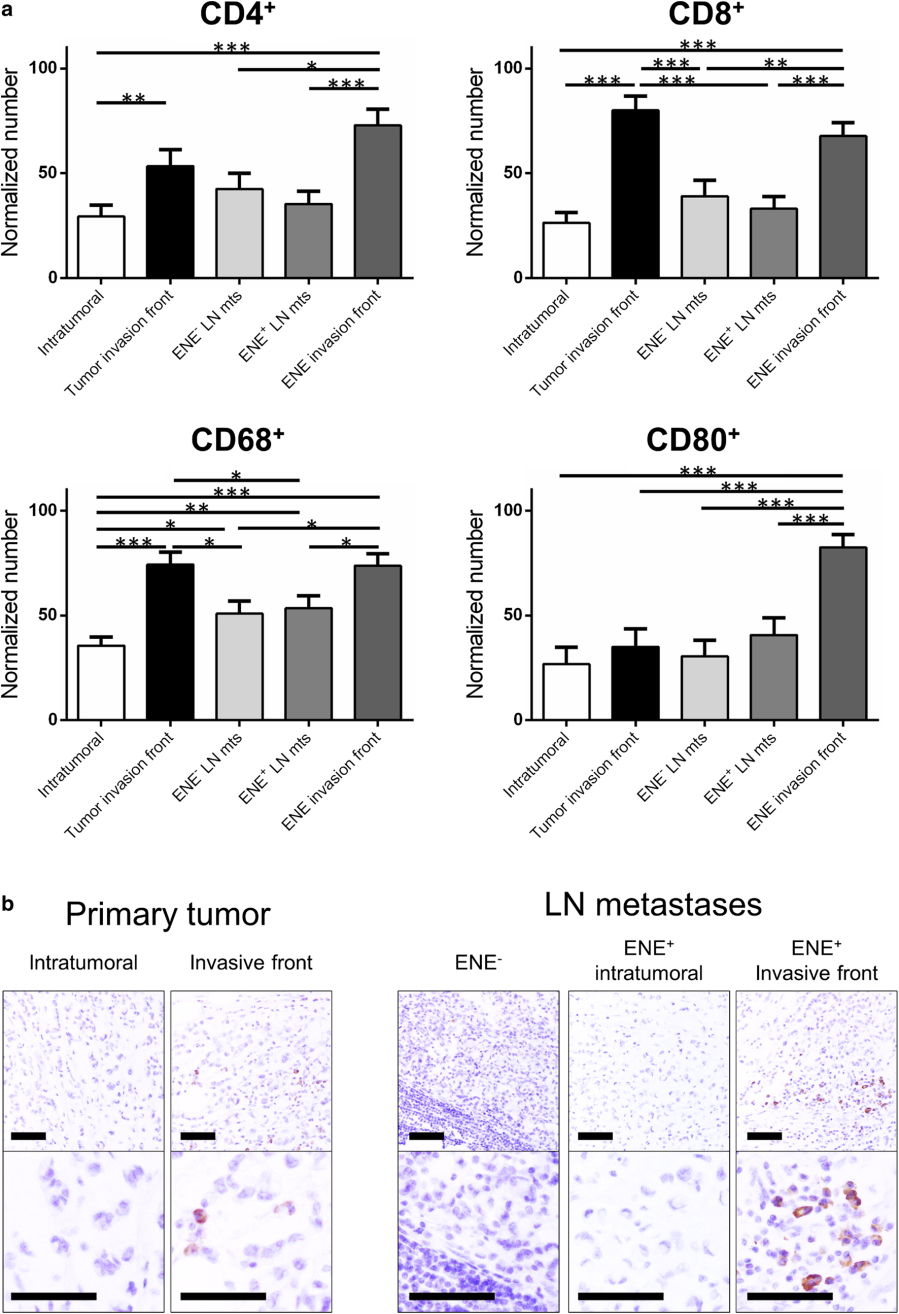
**a** Immunohistochemical characterization of 22 ENE-positive colorectal carcinomas. In each case, stromal positive lymphoid T cells were evaluated in five high-power fields (40×) in different areas of the samples: (i) central part of the primary CRC; (ii) invasive front of the primary CRC; (iii) intratumoral infiltration of the metastatic LN sample; (iv) intratumoral infiltration of the ENE positive LN sample; (v) invasive front of the ENE positive LN sample. A significantly higher percentage of CD4-, CD8- and CD68-positive cells was observed at the invasive front of both CRCs and in ENE in contrast with what observed at the core of both CRCs and their matched nodal metastases. ENE was also characterized by a significantly higher number of CD80-positive cells. (*p < 0.05; **p < 0.01; ***p < 0.001). **b** Representative images of CD80-positive cells distribution within the considered samples (original magnifications 20× and 40×; scale bars = 100 µm)

PD-L1 expression was assessed both in neoplastic epithelia and in infiltrating lymphocytes. The positive neoplastic cells percentage was evaluated jointly by two pathologists. For epithelial immunoreactivity, only moderate/strong membranous staining up to 1% was considered positive. Stromal positive lymphoid cells were evaluated in five HPF (40×) in different areas of the samples: (i) central part of the primary CRC; (ii) invasive front of the primary CRC; (iii) intratumoral infiltration of the metastatic LN sample; (iv) intratumoral infiltration of the ENE positive LN sample; (v) invasive front of the ENE positive LN sample (Fig. 1). Presence of lymph node-like structures at the edge of the primary tumors were not considered in the analysis. Stromal PD-L1 positive cells infiltration of tumors was assessed by semiquantitative estimation of the density of PD-L1-positive cells, and was scored as 0, no positive cell; (1) sporadic PD-L1^+^ cells; (2) moderate numbers of PD-L1^+^ cells; (3) abundant occurrence of PD-L1^+^ cells.

The infiltrating CD4, CD8, CD68 and CD80 leukocytes were counted in five HPF of the previously mentioned five randomly selected tumor areas and final counts were normalized within the same case.

DNA mismatch repair machinery deficient tumors (MMRd) were defined by the absence of nuclear staining in one of the two heterodimers MLH1/PMS2 and/or MSH2/MSH6 in tumor cells, as assessed in the colorectal setting [19].

### Hotspot multigene mutational profiling

DNA was obtained from the FFPE samples after enrichment for neoplastic cells, as previously described with minor modifications [20]. Suitable areas for microdissection were selected from the archival haematoxylin and eosin slides and the corresponding tissue blocks were serially cut to 10-μm-thin sections. Unstained sections were, subsequently, deparaffinized and slightly counterstained with haematoxylin. Tumor cells were dissected manually using a sterile syringe needle, and at least 60% of neoplastic cells were collected from the primary carcinoma and two different metastatic lymph-node, one of which characterized by the presence of ENE. In the ENE-positive lymph-node, only the region with extracapsular extension was considered for microdissection. DNA was extracted using the QIAamp DNA formalin-fixed, paraffin-embedded tissue kit (Qiagen, Milan, Italy) and qualified as previously performed [21].

DNA obtained from the microdissected tumor components underwent hotspot multigene mutational profiling including 164 hotspot regions of the *AKT1*, *APC*, *BRAF*, *CTNNB1*, *KIT*, *KRAS*, *NRAS*, *PDGFRA*, *PIK3CA*, *PTEN* and *TP53* genes using a custom panel (Diatech Pharmacogenetics, Jesi, Italy; primers and protocol available upon request) run on a MassARRAY Dx Analyzer 4 (Agena Bioscience, Hamburg, Germany) [22]. The observed somatic mutations were confirmed by Sanger sequencing (Applied Biosystems 3130xl Genetic Analyser; Life Technologies, Monza, Italy).

### Statistical analysis

The strength of the association between the different histological lesions and the immunohistochemical/molecular features was calculated by applying the Wilcoxon signed-rank test and the Mann–Whitney test, as appropriate. Stata software (Stata Corporation, College Station, TX) was used for all calculations. A *p* value < 0.05 was considered significant.

## Results

### ENE phenotype is characterized by an enhanced inflammatory response at the invasive front

A series of 22 ENE-positive CRCs were analysed by immunohistochemistry for PD-L1, CD4, CD8, CD68 and CD80 expression. For each case, three different samples were considered and analysed: the primary CRC, an ENE-negative and an ENE-positive metastatic LN.

Immunohistochemical analyses showed a significantly higher percentage of CD4-, CD8- and CD68-positive cells at the CRC and ENE invasive fronts in comparison with what observed at the core of both CRCs and their matched ENE-negative nodal metastases (Fig. 1a).

For CD4, the normalized number of positive T-cells was 1.82-fold higher in the CRC invasive front in comparison of the number of positive cells observed at the center of the tumor (*p *= 0.0026; paired *t* test) and 1.71 in ENE invasive front in comparison to the matched ENE-negative metastatic LNs (*p *= 0.029; paired *t*-test).

For CD8, the normalized number of positive T-cells was 3.04-fold higher in the CRC invasive front in comparison of the number of positive cells observed at the center of the tumor (*p *< 0.0001; paired *t*-test) and 1.74 in ENE invasive front in comparison to the matched ENE-negative metastatic LNs (*p *= 0.008; paired *t*-test).

For CD68, the normalized number of positive macrophages was 2.08-fold higher in the CRC invasive front in comparison of the number of positive cells observed at the center of the tumor (*p *< 0.0001; paired *t*-test) and 1.45 in ENE invasive front in comparison to the matched ENE-negative LNs (*p *= 0.015; paired *t*-test).

A significantly higher prevalence of CD80-positive cells was detected in the ENE samples only (Fig. 1a, b). In particular, the normalized number of positive cells was 2.70-fold higher in ENE invasive front in comparison to the matched ENE-negative LNs (*p *< 0.001; paired *t*-test).

No significant number of epithelial, or leukocytic PD-L1 positive cells was observed among the different considered samples.

The four cases with MMRd presented a relatively, but not significant, higher number of CD4 and CD8 infiltrating cells (in both primary and metastatic LNs) in comparison to MMR proficient (MMRp) cases, and were also characterised by a higher number of CD4/CD8 positive cells at both the CRC invasive front in comparison to the center of the tumor and in ENE invasive front in comparison to the matched ENE-negative metastatic LNs.

### No specific mutational signature characterizes the ENE phenotype

Fifteen out of the original series of 22 cases were further profiled through a hotspot multigene mutational custom panel.

**Fig. 2:**
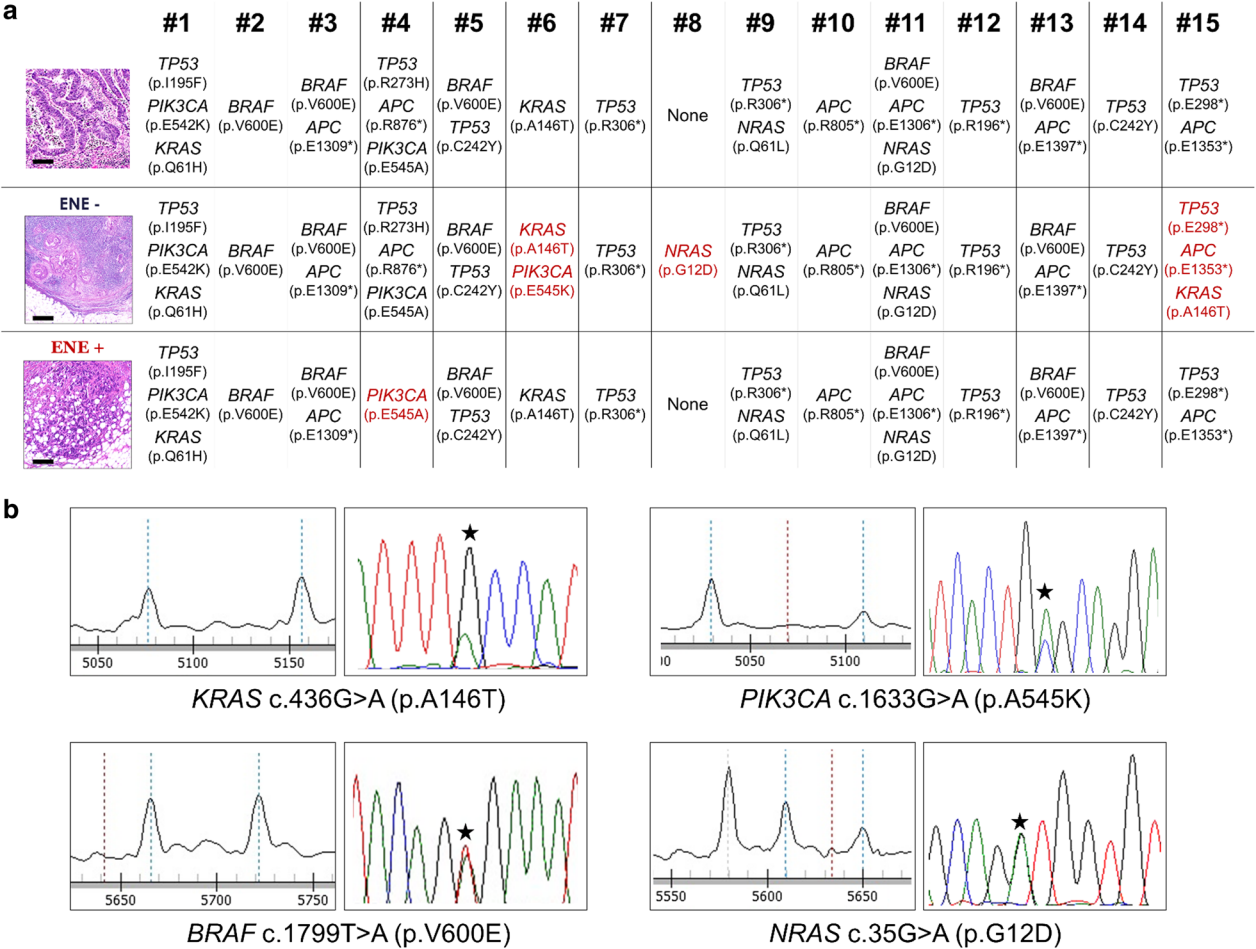
**a** Mutational profiling of the 15 cases profiled through a hotspot multigene mutational custom panel, including 164 hotspot regions of *AKT1, APC, BRAF, CTNNB1, KIT, KRAS, NRAS, PDGFRA, PIK3CA, PTEN* and *TP53* genes. In red are the samples showing a different mutational landscape in comparison to the other matched samples. (scale bars = 100 µm). **b** Representative Sequenom MassArray output profiles for *KRAS* c.436G > A (p.A146T), *PIK3CA* c.1633G > A (p.A545K), *BRAF* c1799T > A (p.V600E) and *NRAS* c.35G > A (p.G12D) mutations. On the right the correspondent Sanger chromatogram

In primary tumors, a total of 25 somatic mutations were observed among the 11 cancer-related genes (Fig. 2). In 14 of 15 (93%) primary CRC samples, at least one somatic mutation was detected; eight lesions were found to have multiple driver gene somatic mutations.

The *TP53* gene was the most frequently mutated gene (8 out of 15), followed by *APC* (six cases) and *BRAF* (four cases; all MMRd). *KRAS*, *NRAS* and *PIK3CA* were mutated in two cases at most. In 11 out of 15 (73%), the molecular profiling of the primary CRC was consistent with that of its matched metastatic LNs, whereas a discrepancy between the mutational profiling of the primary CRC and its matched metastatic LNs was found in four cases (Fig. 2).

The detected allele frequencies of the mutations observed in the four discordant cases was consistent with either polyclonal expansion or monoclonal proliferation of the disease (intra-tumor heterogeneity) in line with recent evidence suggesting that nodal dissemination from primary CRCs is not necessarily related to genetic bottlenecks [23].

No significant association was observed between a peculiar mutational profile and an increase/decrease in inflammatory infiltration.

## Discussion

For many years, the interest in oncological research has focused on understanding the mechanisms that would induce the acquisition of an aggressive phenotype by the neoplastic cells. This may represent the biological rationale in the development of new therapeutics which, on one side, would impair the metastatic ability and, on the other, personalize the oncological and surgical managements of the disease.

Worldwide, CRC is a major cause of morbidity and mortality [1, 2]. The TNM staging system represents the best patient’s prognostic factor at time of the diagnosis. Although the TNM staging system is constantly updated, outcomes of patients with similar histological features falling within the same risk category may have heterogeneous outcomes (i.e. pT3pN1 versus pT4 and/or pN2 in Stage III CRC) thus impacting on clinical decisions [24]. It is, therefore, clear that there is an unmet need to identify new prognostic factors to better stratify the patients, both from a histological and molecular point of view.

The detection of ENE has been demonstrated to be an important prognostic factor in many epithelial cancers [3–14]. Indeed, the latest TNM edition has highlighted the need to report the presence of ENE in lymph node metastasis in case of the head and neck tumors and the vulvar squamous cell carcinoma [25, 26].

Several lines of evidence have proven that, even in CRC, the presence of ENE correlates with a patient’s poor prognosis both in terms of risk of recurrence and overall survival [4, 7, 27–30].

Despite the recognized prognostic impact of the ENE phenotype, no previous work has tried to elucidate its molecular landscape. One of the bottlenecks in this field is the (almost) mandatory use of formalin-fixed paraffin-embedded (FFPE) specimens, which potentially affects many down-stream molecular pathology analyses [31]. Moreover, most of the lesions are small and, thus, their comprehensive molecular characterization is technically challenging.

Starting from archival FFPE material, we investigated: (i) the ENE-associated inflammatory infiltrate by immunohistochemistry; (ii) the ENE molecular background by applying a FFPE-friendly custom panel to characterize 164 hotspot regions of CCR-related genes. Of the initial series of 65 consecutive TNM stages III and IV primary CRC and their matched metastatic LNs, only 22 presented adequate material for the subsequent molecular characterization.

The immunohistochemical evaluation for the presence of tumor-infiltrating T lymphocytes (CD4 and CD8) and macrophages (CD68) demonstrated a significantly higher prevalence of inflammatory infiltrate at the neoplastic invasive front of the primary tumor and of the ENE in contrast with what observed at the core of both CRCs and their matched intra-nodal metastases. These data suggest that there is a common host-related inflammatory systemic response against the infiltrating neoplastic cells. An important negative aspect of this study is the lack of a comprehensive tumor immune microenvironment characterization, which was not possible due to the limited amount of (FFPE) material. Further expression profiling studies should investigate the role of the different tumor areas/tumor-associated stroma in the polarization of the monocyte-macrophage lineage and of the activation of an antitumor milieu.

Of note, high prevalence of CD80 positive cells was observed at the ENE invasive front. This protein is present on dendritic cells, activated B cells and monocytes and its overexpression provides a costimulatory signal necessary for T cell activation and survival. A significant overexpression of CD80 in colonic mucosa has been found in early stages of colorectal carcinogenesis and was demonstrated to play a role in immune surveillance mechanisms in the transition from low-grade to high-grade dysplasia [32, 33]. However, a strong CD80 expression has been associated to resistance to adjuvant chemotherapy suggesting the failure of this specific immune surveillance mechanism in advanced stage of digestive tract cancer [34]. Thus, the association of ENE status with an overexpression of CD80 may be one of the factors which could potentially explain the more aggressive biological behaviour displayed by metastatic CRCs with ENE [16]. The results of this exploratory study call for further investigations about the immunological microenvironment of metastatic lymph nodes with ENE, also for addressing new potential therapeutic strategies.

Even on a relatively small series of cases and by analyzing a limited panel of CRC-associated genes, our study lack to demonstrate any clonal ENE-associated nodal metastases specific molecular driver. Indeed, 73% of the cases show that the mutations which were found in the primary tumors are consistent with those observed in their matched lymph node metastases demonstrating that the tumor clones are still the same (observed for both ENE positive and ENE negative ones). Moreover, no difference was found between the ENE-associated nodal metastases and the ENE-negative one or the correspondent primary tumor in the same patient. This lack of association may be explained with the small sample size of our study, but it is more likely that somatic mutations are not a driver mechanism in the acquisition of the ENE phenotype in CRCs. Recent reports have demonstrated an important clustering of cancer stem-like cells at the CRC invasive front and their important role in the development of tumor heterogeneity [35–38]. In the CRC setting, CD44 variant-positive cancer stem cells have been demonstrated to be resistant to redox stress in the tumor microenvironment by enhancing the ESRP1-CD44v-xCT-GSH (cysteine/glutamate antiporter) axis [35, 38, 39], and thus these cells are almost unaffected by selective pressure determined by oxygen deprivation and anti-cancer treatments Another interaction involving beta-catenin in CRC regards CD200, a membrane protein that characterizes neoplastic cells with cancer stem properties [40]. Due to the important role of CD200 in regulating tumor microenvironment in this setting, its expression may be of importance also in ENE-positive CRCs. Future studies are therefore needed to better define the complete profiles of ENE-positive CRC cell clones.

With our study, we highlight that the biological mechanisms on the basis of ENE in CRCs should be further investigated in the complex immunological microenvironment rather than in the genetic profiles of metastatic cells, including CD44 and CD200. It is also of importance that in other solid malignancies an ENE-specific molecular profile has been detected by comparing ENE-negative and ENE-positive cases [41], but in CRC the importance of metastases microenvironment may be more significant.

## Conclusion

Overall our data demonstrated that there is, on one side, no specific molecular features based on our relatively limited genotypic study for the acquisition of the ENE phenotype but, on the other, the overexpression of CD80 and T lymphocytes/macrophages infiltration at the ENE invasive front may suggest further investigations on a possible application of novel immunotherapeutic approaches in ENE-positive CRCs.

## Abbreviations

CRC: colorectal cancer
ENE: extranodal extension
FFPE: formalin-fixed and paraffin-embedded
MMRd: mismatch repair deficient
MMRp: mismatch repair proficient
TNM: tumor-node-metastasis
