# Correction to: Assessment of intratumor immune-microenvironment in colorectal cancers with extranodal extension of nodal metastases

**DOI:** 10.1186/s12935-019-0966-z

**Published:** 2019-09-24

**Authors:** Matteo Fassan, Luca Vianello, Diana Sacchi, Giuseppe N. Fanelli, Giada Munari, Marco Scarpa, Rocco Cappellesso, Fotios Loupakis, Cristiano Lanza, Roberta Salmaso, Claudia Mescoli, Nicola Valeri, Marco Agostini, Edoardo D’Angelo, Sara Lonardi, Salvatore Pucciarelli, Nicola Veronese, Claudio Luchini, Massimo Rugge

**Affiliations:** 1 Surgical Pathology & Cytopathology Unit, Department of Medicine (DIMED), University of Padua, via Gabelli 61, 35121 Padua, Italy; 2 Department of Surgical Oncology and Gastroenterology (DiSCOG), University of Padua, Padua, PD Italy; 3 Unit of Oncology 1, Department of Clinical and Experimental Oncology, Istituto Oncologico Veneto, IOV-IRCCS, Padua, PD Italy; 4 Division of Molecular Pathology, The Institute of Cancer Research, Sutton, London, UK; 5 Department of Medicine, The Royal Marsden NHS Trust, Sutton, London, UK; 6 Nanoinspired Biomedicine Laboratory, Institute of Pediatric Research, Fondazione Città della Speranza, Padua, PD Italy; 7 Department of Nanomedicine, The Methodist Hospital Research Institute, Houston, TX USA; 8 National Research Council, Neuroscience Institute, Aging Branch, Padua, PD Italy; 9 Department of Diagnostics and Public Health, Section of Pathology, University and Hospital Trust of Verona, Verona, VR Italy; 10 Veneto Cancer Registry, Padua, PD Italy; 11 National Institute of Gastroenterology-Research Hospital, IRCCS “S. de Bellis”, 70013 Castellana Grotte, BA Italy

## Correction to: Cancer Cell Int (2018) 18:13110.1186/s12935-018-0634-8

Following publication of the original article [1], it has been brought to our attention that an incorrect Sequenom MassArray trace and an incorrect nomenclature were used to represent the PIK3CA p.E545A mutation in Fig. 2b. The correct Fig. 2b is shown in this erratum. The authors apologize for the confusion.

**Fig. 2:**
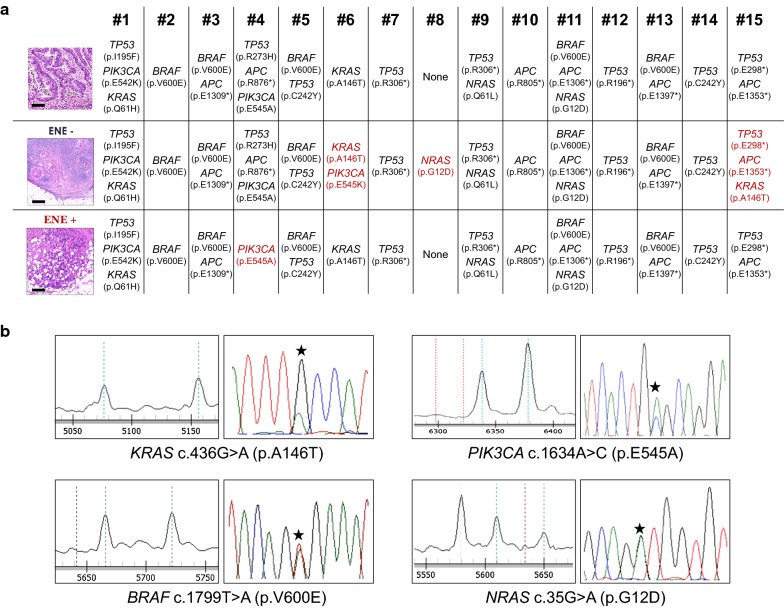
**a** Mutational profiling of the 15 cases profiled through a hotspot multigene mutational custom panel, including 164 hotspot regions of *AKT1, APC, BRAF, CTNNB1, KIT, KRAS, NRAS, PDGFRA, PIK3CA, PTEN* and *TP53* genes. In red are the samples showing a different mutational landscape in comparison to the other matched samples (scale bars = 100 µm). **b** Representative Sequenom MassArray output profiles for *KRAS* c.436G> A (p.A146T), *PIK3CA* c.1634A>C (p.E545A), *BRAF* c1799T>A (p.V600E) and *NRAS* c.35G>A (p.G12D) mutations. On the right the correspondent Sanger chromatogram
